# Supermicrosurgical Vascular Anastomosis—A Comparative Study of Lumen-Enhancing Visibility Techniques

**DOI:** 10.3390/jcm14020555

**Published:** 2025-01-16

**Authors:** Vladut-Alin Ratoiu, Andrei Cretu, Florin-Vlad Hodea, Catalina-Stefania Dumitru, Andreea Grosu-Bularda, Eliza-Maria Bordeanu-Diaconescu, Razvan-Nicolae Teodoreanu, Ioan Lascar, Cristian-Sorin Hariga

**Affiliations:** 1 Department 11, Discipline Plastic and Reconstructive Surgery, Clinical Emergency Hospital of Bucharest, “Carol Davila” University of Medicine and Pharmacy, 050474 Bucharest, Romania; vladut-alin.ratoiu@rez.umfcd.ro (V.-A.R.); florin-vlad.hodea@drd.umfcd.ro (F.-V.H.); catalina-stefania.dumitru@rez.umfcd.ro (C.-S.D.); andreea.grosu-bularda@umfcd.ro (A.G.-B.); razvan.teodoreanu@umfcd.ro (R.-N.T.); ioan.lascar@umfcd.ro (I.L.); cristian.hariga@umfcd.ro (C.-S.H.); 2 Clinic of Plastic Surgery and Reconstructive Microsurgery, Clinical Emergency Hospital of Bucharest, 014461 Bucharest, Romania; eliza.diaconescu@umfcd.ro

**Keywords:** microsurgery, supermicrosurgery, non-living model, simulation training, techniques, double-contrast

## Abstract

**Background**: The development of microsurgical techniques has enabled reconstructive versatility in various clinical scenarios. Supermicrosurgery is an advanced microsurgical technique ensuring precise reconstructions by operating on small-caliber vessels and nerves, with applications in reconstructive surgeries. **Objectives**: This study aims to compare the effectiveness of four low-cost training models, thereby improving surgical precision and reducing the learning curve for novice surgeons. **Materials and Methods**: We conducted a prospective non-randomized study comparing the classic anastomosis technique, the intravascular stenting (IVaS) technique, the color contrast (CC) technique, and our newly introduced double-contrast (DC) technique, which combines IVaS with CC. We used a non-living experimental model represented by chicken wings, analyzing the vessel preparation and anastomosis time, anastomosis patency, and back wall biting through a standardized protocol. We performed 120 end-to-end anastomoses in total, with vessel diameters ranging from 0.5 to 0.8 mm. **Results**: CC demonstrated superior time efficiency and success rates, reaffirming it as a reliable option in supermicrosurgery, while DC showed slightly better time efficiency and patency compared to both IVaS alone and the classic method. CC outperformed the others in anastomosis time, patency, and back wall catching, reaffirming its reliability in supermicrosurgery. **Conclusions**: Although DC did not significantly improve patency, it reduced back wall engagement. This makes the DC technique beneficial for trainees working on vessels under 0.5 mm in diameter, where stenting is often required, improving surgical precision and reducing the learning curve, leading to better outcomes in supermicrosurgery.

## 1. Introduction

Microsurgery refers to a set of techniques that utilize optical magnification and specialized micro-instruments to perform precise operations on small anatomical structures [1]. Microsurgery has become an indispensable component of the surgical repertoire across numerous subspecialties, including plastic surgery, neurosurgery, transplant surgery, ophthalmology, otolaryngology, and even urology [1,2,3].

In plastic surgery, microsurgical techniques are an essential component of the reconstructive surgical spectrum, forming the basis for procedures such as replantations, free autologous tissue transfer, peripheral nerve repair, reconstruction, and lymphatic surgery. The evolution of modern microsurgery is closely linked to advancements in microscopes and micro-instruments. In the 1950s and 1960s, the first clinical applications of microsurgery were implemented in limb replantation through the anastomosis of small-caliber arteries, veins, and peripheral nerves. Meticulous microsurgical repair of vessels and peripheral nerves in trauma cases is crucial for restoring the functionality of the replanted segment [1,3,4,5,6].

Another area of significant interest is the reconstruction of defects resulting from tumor excisions. These cases often involve complex, multi-layered excisional defects for which the adjacent tissue resources are insufficient to provide adequate coverage. Additionally, functional restoration is frequently a key objective in such reconstructions [7,8,9].

The use of radiotherapy as a therapeutic modality introduces potential complications and challenges in developing an effective reconstructive plan for oncology patients. Complex procedures, such as post-mastectomy breast reconstruction, advanced reconstructions following head and neck tumoral excisions, and sarcoma resections, require precise surgical techniques performed by a well-trained microsurgical team [10,11,12,13,14].

Ongoing advancements in microsurgery, including the development of supermicrosurgical techniques and the integration of robotic assistance, have greatly expanded the possibilities and improved outcomes in plastic surgery [1].

Supermicrosurgery is a highly specialized field in microsurgery that involves the dissection, manipulation, and anastomosis of considerably small vessels and nerve fascicles, typically ranging from 0.3 to 0.8 mm in diameter. This advanced technique requires the use of powerful microscopes, delicate instruments, and proper training for microsurgeons in the field in order to achieve efficient outcomes. Supermicrosurgery has revolutionized the field of reconstructive surgery by enabling procedures that were previously unattainable, such as true perforator flap surgeries, lympho-venous anastomoses (LVAs), and vascularized lymph node transfers (VLNTs) [15,16,17].

The applications of supermicrosurgery are diverse and expanding. It is commonly used in reconstructive surgeries, including fingertip reconstructions and craniofacial and head and neck delicate soft tissue reconstructions. Another important field in which supermicrosurgery is an essential skill is represented by lymphedema, which is a severely debilitating condition and a common complication following the treatment of different malignancies or less common underlying congenital diseases, infections, trauma, or radiation [18]. For some patients who suffer from chronic lymphedema, conservative therapy sometimes proves to be effective alone; however, other patients may benefit from combined surgical and conservative treatment [19]. Thus, the role of supermicrosurgery in treating lymphedema has gained significant attention, as surgeons can now create new pathways for lymphatic drainage, improving patient outcomes and quality of life [20].

The primary technical challenges encountered in supermicrosurgery, particularly concerning microvascular anastomoses, stem from the intricacies associated with the thin-walled structure and the small diameter of the vessels involved. These characteristics significantly restrict the application of traditional forceps during intravascular manipulation of the needle. Consequently, this limitation heightens the risk of inadvertently engaging the back wall of the vessel, which can lead to critical complications, including vessel wall injury and, ultimately, thrombosis. To address these concerns, a variety of innovative methods have been developed to mitigate the risk of unintentional back wall catching and to safeguard the integrity of the vessel wall during supermicrosurgical procedures [21].

Furthermore, the training required to achieve proficiency in supermicrosurgery is tedious and resource-demanding. There is a need for specialized training programs and simulation models to help surgeons develop the necessary skills for anastomoses of structures under 1 mm. The limited availability of clinical cases and the high cost of simulation resources complicate the training process [22].

In our study, we aimed to rigorously compare the performance and outcomes of a low-cost, reproducible, non-living training model using four techniques: the standard or classic surgical method (without any staining or stenting), the intravascular stenting technique (IVaS, where a stent is placed intraluminally), the color contrast technique (CC, where the vascular edges are stained with a colored marker), and lastly a combination of IVaS and CC, further named throughout the manuscript as the double-contrast technique (DC) [23,24]. Our aim is to discover which one is the most efficient in terms of enhancing surgical precision, thereby minimizing the learning curve experienced by novice surgeons. Thus, as the primary objective, we aim to assess the efficiency of the four techniques in ensuring a correctly performed anastomosis, which is the key element in the success of a microsurgical procedure. This comparison is expected to translate into superior outcomes during supermicrosurgical procedures, which require a high degree of skill and accuracy. Secondarily, we aim to develop a suitable training model that allows a young surgeon to improve their microsurgical skills and practice very fine techniques, as well as ensuring a smooth translation to further experimental living models and ultimately clinical practice. By providing a structured and effective training model, we believe that the results of this comparative study, measured through the rates of patency, back wall biting, and time for anastomosis completion, could greatly benefit the surgical community, particularly in the context of complex vascular repairs and interventions.

## 2. Materials and Methods

A prospective non-randomized study was designed and performed, in which four different supermicrovascular techniques were compared using the unanimously accepted non-living model of chicken wings in the Experimental Microsurgery Laboratory of the Plastic and Reconstructive Surgery Clinic in the Clinical Emergency Hospital Bucharest, Romania, based on previously established models [25].

A standardized protocol was established and adapted after the non-living model available in the literature [26]. All anastomoses were performed by a single surgeon, a resident in training, on non-living commercially acquired models (ex vivo chicken wings). The surgeon was a second-year resident (the equivalent of a PGY-2 training level) at the time of this study who had completed their 6-month general surgery rotation. In addition to participation in a microsurgical training course, the surgeon had clinical experience, taking part in microsurgical interventions, which were mainly focused on trauma cases. The training in our clinic starts in the experimental laboratory for each resident at the beginning of the second year, following a stepwise protocol starting from non-living models and increasing in complexity to include latex gloves, leaves, flower petals, silicone tubes, and chicken thighs [25]. Further on, the trainee practiced on living animal models represented by Wistar rats, including working on the femoral and carotid arteries. All these steps were completed by the resident who performed the anastomoses described in this study.

One hundred and twenty fresh chicken wings were dissected in order to find the ulnar artery. The skin was resected and the dissection was carried out from the wrist crease up to the wing tip. The ulnar artery was discovered under the fascia, between the flexor carpi ulnaris muscle and the flexor digitorum superficialis muscle of the chicken wings, along with the concomitant vein. Blunt dissection was preferred to prevent injuries to the arterial wall. The artery was cleared of fat and adjacent connective tissue, and the communicating branches were ligated to allow better vessel mobilization. The selected vessels included in this study were under 0.8 mm in diameter. Then, the artery was divided, the adventitia was trimmed, the vessel was dilated using a 0.1 mm vessel dilator (Aesculap, Tuttlingen, Germany), and the lumen was washed out with a heparinized solution until clear. The flow through the “blue blood” system, a methylene blue-colored saline solution, was simultaneously installed, as described by Zeng et al [27].

**Figure 1:**
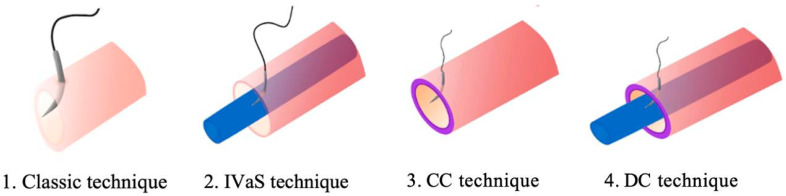
Graphic drawings representing the four techniques: 1—classic technique, 2—intravascular stenting technique, 3—color contrast technique, 4—double-contrast technique. Blue – intravascular stent; Purple – gentian violet staining.

The anastomosis was alternatively performed using the four different techniques, as illustrated in Figure 1.

**Figure 2:**
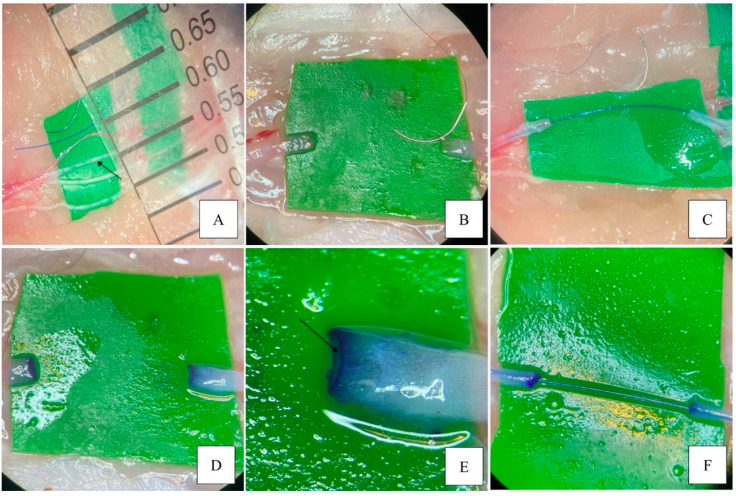
Dissection and techniques illustration. (**A**) Chicken wing dissection with exposure of ulnar artery (arrow) and vessel selection by size (<0.8 mm). (**B**) Standard classic microvascular anastomosis technique. (**C**) IVaS anastomosis technique. (**D**) CC anastomosis technique. (**E**) Close-up of the CC technique, clearly showing the contrast and good visibility inside the lumen (arrow). (**F**) DC anastomosis technique.

As shown in Figure 2, the methods used were the standard or classic method (in which we used neither a stent nor any markings on the vascular wall), IVaS (stenting of the vessel), CC (gentian violet staining of the vessel), and DC (combining gentian violet staining of the vessel ends with IVaS). The stent size was standard polypropylene 5/0 and the stent length ranged from 10 to 20 mm. We first introduced the stent into the proximal end and then into the distal end of the vessel. The anastomoses were performed using nylon and polyamide 10/0 sutures with round-tipped needles (Ethicon, USA; Demophorius, Cyprus), which were selected not only due to their availability in the laboratory, which ensured the replicability and uniformity of this study, but also because of their ease of handling compared to finer alternatives, which facilitated the experimental procedure. We did not use a microvascular approximator in any of the anastomoses. After the placement of the first suture, the stent was advanced proximally inside the vessel, leaving a shorter portion of the stent in the distal end. The first two sutures were placed on opposite sides of the vessel. After completing the front wall, the vessel was turned over for the back wall anastomosis. For the final two sutures on the back wall, the open-loop technique, also known as the running interrupted technique, was used in order to prevent accidental entrapment of the back wall and for a better view of the lumen. The stent was carefully moved proximally inside the vessel and withdrawn with its distal end through the space left by the open-loop technique. Finally, the open-loop sutures were tightened after a final check of the lumen. 

The vessel’s external diameter, the time required for vessel preparation, and the anastomosis were registered together. Back wall bites were evaluated, and anastomosis patency was tested using two main methods. The first one is the aforementioned “blue blood” system, which was installed proximally to the anastomotic site of the vessel, allowing the evaluation of both patency (when the blue saline traveled through the anastomosis and further distally) and anastomotic leakage (when the blue saline poured from the anastomotic site into the surrounding tissues). The second way of assessing the anastomosis was through direct inspection under a microscope, after cutting the vessel proximally and distally to the anastomotic site and then longitudinally, so that visual access to the intimal layer was possible. Since this study involved a non-living mechanical model, vascular wall damage was not tracked as a decisional parameter within the final database to influence the duration or patency. Further analysis of wall damage was not applicable, and pathological microscopic tests for vessel analysis were not performed.

Data were gathered from each anastomosis and the results were recorded in digital databases and analyzed in comparison to current medical papers found on the PubMed and Google Scholar search engines. The quantitative variables included in this study were vessel diameter and the time taken for anastomosis completion (including vessel preparation), whilst the recorded qualitative variables were the presence of back wall biting during the procedure and the final patency of the anastomosis. The variables were analyzed using Microsoft Excel version 16.77.1 and Minitab^®^ Statistical Software (Cloud App & Windows Desktop). A chi-squared test was used to establish if the null hypothesis was true when comparing tested techniques for qualitative variables, while quantitative variables were assessed using the *t*-student test (*p*-value considered significant under 0.05). All statistical tests were applied after confirming the normal distribution of data using the Anderson–Darling test.

## 3. Results

We performed a total of 120 end-to-end anastomoses on different levels of the ulnar artery in chicken wings, alternating equally between the four different techniques described above as follows: 30 classic anastomoses, 30 IVaS anastomoses, 30 CC anastomoses, and 30 DC anastomoses.

Vessel diameters ranged from 0.5 mm to 0.8 mm. The vessels’ mean diameter was 0.652 mm, with a median of 0.65 mm and a standard deviation of 0.08 mm. When separating each technique, the mean diameter for the classic technique was 0.67 mm, with a median of 0.7 mm and a standard deviation of 0.073 mm, the mean diameter for IVaS was 0.66 mm, with a median of 0.675 mm and a standard deviation of 0.079 mm, the mean diameter for CC was 0.64 mm, with a median of 0.65 mm and a standard deviation of 0.088 mm, and the mean diameter for DC was 0.62 mm, with a median of 0.65 mm and a standard deviation of 0.077 mm. There was no statistical difference between the vessel dimensions of each group.

Overall, the patency rate was 87% (104 out of 120 anastomoses were patent). For both the classic technique and IVaS, the patency rate was 80% (24 out of 30 anastomoses were patent), while for CC, it was 100% (30 out of 30 anastomoses were patent) and for DC, it was 87% (26 out of 30 anastomoses were patent). However, back wall bites were observed in only 25% of the failed anastomoses (four out of the sixteen non-patent anastomoses), all of which were in the classic method group, meaning that no back wall biting was observed in the IVaS, CC, and DC groups (Table 1).

We found several statistical differences between the groups concerning patency (Table 2), such as when comparing CC with the classic technique (*p* = 0.009), with IVAS (*p* = 0.009), with DC (*p* = 0.03), and with non-staining techniques (*p* = 0.008). No statistical differences regarding patency were found between the classic technique, IVaS, and DC (*p* > 0.05).

After grouping IVaS and DC, as both involve the use of an intraluminal stent, we reanalyzed the same variables, without any difference between them and the classic technique. The analysis showed a significant difference between the patency obtained with the stenting techniques versus CC (*p* = 0.01). The stenting techniques were also compared to the non-stenting techniques together, without any statistical significance (*p* > 0.05). Using a similar rationale, we then grouped CC and DC, as they both use vessel staining, with higher patency than the non-staining groups together (*p* = 0.03), but not individually (*p* > 0.05). Lastly, a higher patency rate was observed for CC compared to all of the other techniques together (*p* = 0.01).

Due to the lack of overlapping results between patency and back wall biting, the chi-squared test was applied separately for these two variables. Thus, when the rates of back wall stitching were analyzed, there was a statistically significant difference (*p* = 0.03) between the classic technique and each of the other three techniques separately. No differences regarding back wall biting were found between IVaS, CC, and DC (*p* > 0.05).

Moreover, there was a strong statistical difference regarding back wall biting between the classic approach and both stenting techniques taken together (*p* = 0.003), as well as an even stronger difference between the classic approach and the other techniques altogether (*p* = 0.0004). Finally, back wall biting was also found to be statistically different between the two staining groups and the two non-staining groups (*p* = 0.04).

Microscopic visual examination revealed no apparent signs of arterial wall injury associated with stenting techniques, such as mechanical deformation, intimal detachment, or partial stripping.

The overall average time required to perform one anastomosis was 19:38 min, with a median time of 19:01 min. When separating each technique, the overall time values for the classic technique, IVaS, CC, and DC were 20:42 min, 21:15 min, 15:40 min, and 20:55 min, respectively. The median times for the classic technique, IVaS, CC, and DC were 20:32 min, 21:07 min, 15:24 min, and 20:45 min, respectively (Table 3).

**Figure 3:**
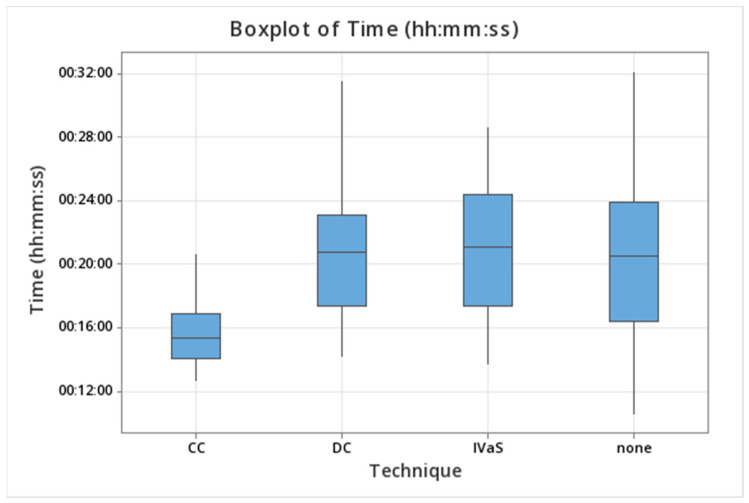
Boxplot representation of the time required for anastomosis for each technique, proving a visible advantage for the color contrast technique.

After analyzing these data, we found that CC was the fastest one of the four (Figure 3), which is true both when compared with the other three methods separately, altogether, or just with the two stenting techniques, with all comparisons returning *p*-values < 0.0001. Although the staining methods together were statistically faster than both the classic technique (*p* = 0.03) and IVaS (*p* = 0.004), there were no statistical differences when comparing DC separately with either one. No differences in the time required for anastomosis were found when comparing IVaS and the normal technique, or when analyzing the two stenting techniques against the classic one. However, statistical differences were observed when comparing non-stenting techniques with the stenting techniques (*p* = 0.0006) and when comparing staining techniques with non-staining techniques (*p* = 0.001).

## 4. Discussion

Over the past six decades, microsurgery has experienced significant evolution, starting with revascularization and replantation techniques and progressing to free microsurgical tissue transfer for addressing complex tissue defects and employing composite tissue transfer methods. The advent of supermicrosurgical techniques has further revolutionized the field, playing a crucial role in the microsurgical treatment of lymphedema and the reconstruction of tissues using perforator flaps. Proper training is essential for successfully implementing these complex techniques in clinical practice [4,16,28].

Although microsurgery is a fundamental and widely practiced technique among plastic surgeons, supermicrosurgery is a complex skill to train in and apply, which can pose challenges when dealing with extremely thin and small vessels [23]. Among the first to attempt to use a form of stenting-based microvascular suture was Wei et al. in 1982, relying on a silastic tube for the anastomosis of the femoral vein in rats [29]. It is similar to the IVaS method as it allows for easy inspection of the lumen and prevents back wall catching. Turning a vessel to suture the back wall can cause lumen deformation, whereas the IVaS technique preserves the vessel lumen’s position and shape, allowing for accurate suturing [23].

The IVaS method offers significant advantages in surgical applications, primarily due to its use of monofilament sutures such as polypropylene or nylon. These materials are particularly beneficial as they provide a flexible range of sizes tailored to accommodate the varying diameters of different vessels. This adaptability ensures optimal fit and support, which is crucial for maintaining the integrity of vascular structures during procedures. However, despite reducing the risk of intimal injury by catching the wall in the suture, stenting the vessel poses a higher risk of wall injury by handling the vessel when removing the stent. This risk can be diminished by cutting the end of the stent with a surgical knife instead of scissors in order to produce a clean cut with a smooth end [23].

Moreover, the choice of color in these sutures, typically blue or black, enhances visibility during surgery. The contrasting hues allow for easier identification against the surrounding tissue, facilitating precision in placement and reducing the risk of complications [30].

The IVaS technique has been adapted in various studies throughout the literature, with different studies implementing different changes to the original technique in order to improve it and to achieve better results.

The bungee-IVaS technique involves securing a 9-0 nylon suture to the stent, leaving a long end to serve as the bungee cord extension. Once this extension is firmly in place, the stent is carefully inserted into the two ends of the vessel, so the anastomosis is performed using the conventional IVaS technique. When ready for removal, microsurgical forceps are gently used to pull the bungee cord, allowing the stent to dislodge smoothly without the need for intraluminal instruments [31].

The preparatory intravascular stenting technique (PIST) is a very good technique in cases of early-stage lymphedema when the vessel caliber is less than 0.3 mm and the vessel can easily collapse. In this technique, a 9-0 nylon thread is first inserted into the target lymphatic vessel. With the nylon in place, the vessel is then transected, and the lumen is stabilized [32]. Even though larger vessels were used, we applied the same stent extraction technique.

The hemi-IVaS technique is performed by inserting an intravascular stent into one side of the vessel, which proves very useful when there is a vessel mismatch. In comparison to hemi-IVaS, we performed the anastomosis on vessel ends with the same caliber; therefore, we believe IVaS to be easier and more useful when there is no mismatch [33].

The double flipping technique involves inserting a 7-0 nylon suture into each side of the blood vessel to prevent occlusion. Double-vein clamps are then applied to the proximal and distal ends of the vessel, securing them along with the nylon. The forceps can then be used to grasp the nylon, allowing for careful manipulation of the vessel lumen. This technique can potentially deform the lumen due to the flipping process, whereas the IVaS technique preserves the shape of the lumen with the stent. The IVaS technique may be more complex due to the stent insertion, but it offers a more stable and consistent approach, particularly for delicate vessels [34].

The temporary assisting suspension suture (TASS) technique involves placing temporary assisting suspension sutures in the donor and recipient arteries, first at the 12 o’clock position and then at the 4 o’clock and 8 o’clock positions. The TASS technique is a valuable method in microsurgery, providing several benefits, such as enhanced vessel alignment, reduced intraoperative manipulation, a minimized risk of lumen deformation, stabilization during suturing, and improved visualization [29].

We tried to further enhance visibility during the process of supermicrovascular anastomosis by employing the DC technique. A comparative analysis of the techniques is shown in Table 4.

Commonly used vessel marking dyes are mainly represented by methylene blue and gentian violet. It is well-known that methylene blue inhibits cyclic guanosine monophosphate (GMPc), therefore abolishing the antithrombotic effect of endothelial nitrous oxide (NO). Some reports in the literature have studied the mechanism and concluded that it is a GMPc inhibitor that does not interfere with NO synthesis [35,36]. On the other hand, some recent studies concluded that methylene blue treatment is linked to a reduction in the thrombin-generating capacity of plasma, but it has minimal impact on the strength of clot formation [37].

Gentian violet is a cationic triphenylmethane dye that is used in some countries as the standard dye for surgical marking pens [38]. Because of its antibacterial and antifungal properties, gentian violet has had various medical and veterinary applications. The evidence of carcinogenicity of gentian violet was discovered in two studies. Animals that had been orally exposed died as a consequence of hepatocellular carcinoma development and histiocytic sarcomas of the bladder, ovary, uterus, and vagina in females [39]. Another recent study discovered that gentian violet significantly elevated reactive oxygen species (ROS) levels and increased the expression of p53, PUMA, BAX, and p21, key apoptosis-related proteins in ovarian cancer cells. These findings suggest that it has potent antiproliferative properties and could be a promising candidate for further investigation as a therapeutic agent for ovarian cancer [40].

Gentian violet colors the vessel layers in different hues: a darker shade of violet on the media and a lighter shade on the intima. We consider that minimal staining of the vessel edges may not generate a pro-oncogenic effect; however, this aspect may still need clinical validation. Further research is warranted to elucidate the mechanisms underlying these effects and to determine the optimal strategies for clinical application.

Supermicrosurgery requires a higher skill level for eye–microscope–hand coordination, more dexterous handling of tissues, and more refined motor skills than microsurgery. To successfully perform these technically demanding procedures, it is essential to develop and practice the required technical skills, first by using non-living models and only then using living animal models. Although living animal models provide vessels of appropriate caliber for supermicrosurgical training, these models are costly and require dedicated research programs, ethical protocols, care protocols, and pre- and postoperative care [26].

Synthetic models are recommended for developing fundamental supermicrosurgical skills. The simplest models include practice cards with silicon tubes, which can be used for practicing the anastomosis of supermicrosurgery-caliber vessels [16,41]. Higher-fidelity biological cadaveric models, such as the chicken thigh model, are suitable for advanced skill acquisition after the trainee has mastered basic skills and is proficient in using the microscope and related instruments. Inspired by the chicken thigh model introduced by Chen et al., we believe that it is more convenient to use chicken wings because the ulnar artery is easier to find. A significant advantage of non-living biological models is that they provide tactile feedback that is almost identical to that experienced in human procedures. Additionally, these models are cost-effective, accessible, and effective [25,26].

In our study, unintentional biting of the back wall and subsequent anastomosis failure occurred exclusively with the classic standard method. All four failures occurring after using the classic technique (13.33%) were attributed to accidental snagging of the back wall. On the other hand, none of the anastomosis failures in the IVaS and DC groups were attributed to back wall biting.

The results of our study showed statistical significance when comparing the classic technique with each of the other three, IVaS, CC, or DC (*p* = 0.03), all of which proved to be superior in this regard. We then grouped the two stenting techniques (IVaS and DC) and compared them with the classic method (*p* = 0.003), showing an even stronger statistical significance. Furthermore, when it comes to back wall stitching, the classic technique was shown to be inferior to the other three combined, with the strongest statistical significance (*p* = 0.0004). When the vessel is neither colored nor stented, the intraluminal visibility is reduced, especially in small-caliber vessels, because there is barely any visual difference between the intima and the media. Hence, we concluded that the probability of catching the back wall when interposing a stent between the vessel walls is diminished by the deflection of the needle in its trajectory toward the back wall.

**Figure 4:**
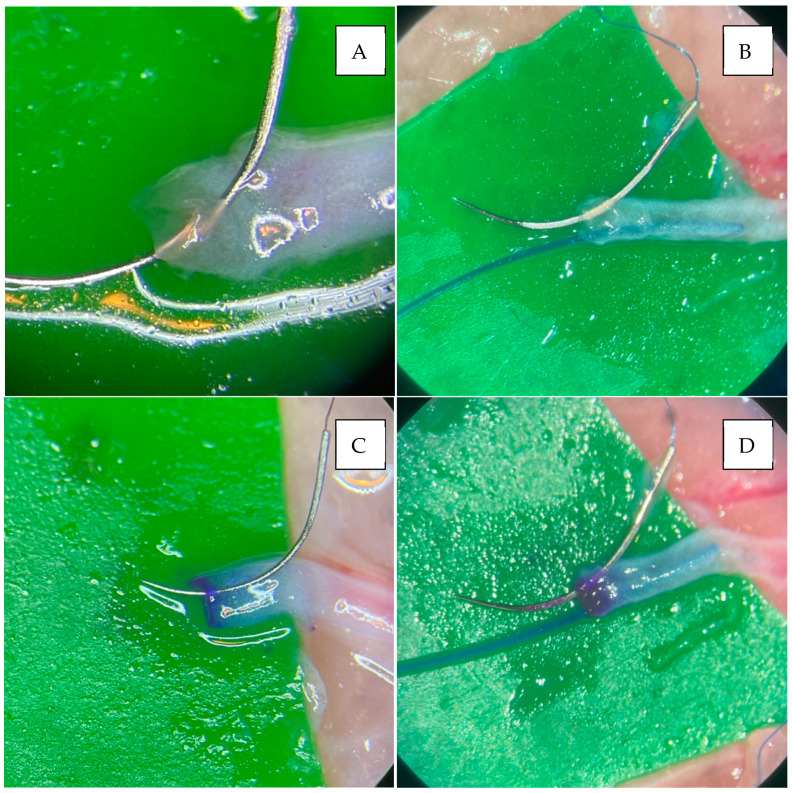
Close-up shots of the needle’s passage through the vessel wall. (**A**) Back wall stitch in the classic technique, where visibility inside the lumen is reduced. (**B**) Correct passage of the needle in IVaS, with the stent clearly visible inside the lumen. (**C**) Correct passage of the needle in CC, with a clear color gradient between the sides of the vessel wall. (**D**) Correct passage of the needle in DC, with the stent clearly visible inside the lumen and a clear color gradient between the sides of the vessel wall.

There was also a significant superiority of the staining groups (CC and DC) compared to the non-staining techniques (*p* = 0.04) regarding back wall stitching. However, since there was no statistical difference between the IVaS, CC, and DC groups individually, we presume that this difference came from the inferiority of the classic technique alone. An interesting observation, though, is that CC proved to be equivalent to the stenting techniques, which makes the staining of the vessel similar to the intraluminal stent in terms of efficacy in preventing back wall biting. These observations highlight the importance of using adjuvant techniques, such as an intraluminal stent or vessel staining, to avoid back wall stitching (Figure 4). However, we emphasize that these results were gathered on a sample of vessels with diameters of 0.5 to 0.8 mm, and we hypothesize that, in even smaller vessels (<0.5 mm), stenting techniques might prove to be superior, not just equivalent, to color contrast alone, although this must be investigated in further studies.

A lack of patency was present in the classic, IVaS, and DC groups, with six (20%), six (20%), and four (13%) anastomotic failures, respectively. No anastomotic failures were present in the CC group. Statistical significance was not achieved regarding anastomotic patency between the classic technique, IVaS, and DC, not even when comparing the stenting methods altogether against the classic one or against both non-stenting techniques. The primary reason for the failure of stented anastomoses is likely the traumatic manipulation of the vessels during stent placement, especially during stent removal.

Despite cleanly cutting the stent with a blade, the contact with the intimal layer and, moreover, the friction between them, may be the cause for vessel injury. A rather proximal placement of the stent at the beginning of the anastomosis, leaving a shorter length in the distal vessel end, was applied in our study in order to diminish this downside of the technique by limiting the amount of friction between the stent and the intimal layer. Another detail of the technique that aims to reduce injury by the stent is the implementation of the open-loop technique for the last two suture points, thus providing sufficient space in the anastomosis through which the stent can be safely removed. We did not use a vascular approximator in this study in any of the techniques, taking into consideration that pressure applied in stenting techniques with a vascular clamp can further provoke vessel wall lesions. However, taking into consideration the lack of back wall stitching in the non-patent anastomoses using the stenting techniques, we conclude that a perfectly atraumatic technique was impossible to achieve with a stent. Thus, we observe that even though IVaS and DC are statistically protective against back wall biting, they are not sufficient in order to improve overall anastomotic patency rates.

Patency is where CC showed significant superiority when compared to the classic technique or IVaS (*p* = 0.009), to DC (*p* = 0.03), to the stenting techniques (*p* = 0.01), and to all the other techniques combined (*p* = 0.01). Moreover, when the two staining techniques were grouped, they showed higher patency than the two non-staining groups (*p* = 0.03), proving the superiority of the CC group, since there were no differences between the classic method, IVaS, and DC. Hence, staining the vessel offers enough visibility inside the lumen to enable the surgeon to make confident gestures and movements. This marked superiority of CC comes from having the same efficacy in preventing back wall stitching as an intraluminal stent, but without the mechanical and traumatic disadvantages of one.

Differences in average times are negligible (*p* > 0.05) between the classic technique, IVaS, and DC. Stenting techniques combined also proved to be nearly as fast as the classic method, which cannot be said about the staining techniques, which were significantly faster than the classic method (*p* = 0.03), IVaS (*p* = 0.004) and both non-staining techniques (*p* = 0.001). However, once again, CC proved to be the superior method among the four, with faster anastomosis times than all other three techniques separately or altogether, and was faster than the stenting methods combined (*p* < 0.0001). Moreover, non-stenting techniques proved to be faster than stenting techniques (*p* = 0.0006), with this being logically explained by the fact that it takes additional time to insert an intraluminal stent. In addition, maybe even more relevantly, it takes additional time to extract it at the very end of the anastomosis through a very narrow space between the vessel ends.

For vessels with slightly larger calibers than those that we used (in the higher range of supermicrosurgery, between 0.5 mm and 0.8 mm) or in less complex cases, the classic technique may still be sufficient, although it has higher risks of failure due to back wall injury, meaning that CC can be employed to maximize results.

The significantly higher time efficiency of the CC technique, combined with a high success rate, proves that it is a reliable method in supermicrosurgery, which is consistent with other findings in the literature [42] and makes it particularly advantageous in clinical settings where time efficiency is a critical factor. Though simpler than stenting methods, it effectively prevents back wall injury through staining, making it competitive with stenting techniques without the additional risks regarding vessel wall damage. CC may be suitable for digital replantations or revascularizations, where the vessel wall is already damaged by the traumatic agent and, despite correct debridement of the injured vessel, an intravascular stent may further enhance the risk of thrombosis. However, CC may be less appropriate for extremely small or fragile vessels where stenting provides more structural support.

The DC technique has its own advantages, namely good visibility of the lumen of thin and damaged vessels and a reduced risk of inadvertent catching of the back wall of the vessel. Moreover, it helps to maintain a proper distance between the sutures and the vessel edges, it is a reasonable method when the vessel has a thin or damaged wall, and it is cost-effective since it does not require sophisticated materials. On the other hand, DC has certain disadvantages as well. Stent insertion into both sides is technically harder and requires more experience, and careful control of the stent is needed because it can be easily removed from the lumen, requiring reinsertion. Lastly, there is a risk of adverse reactions depending on the contrast agent. Even though we proved that the DC technique might not be as optimal as CC, it has a marginally higher time efficiency than the classic method and IVaS and a reasonable patency rate, suggesting that it might have potentially beneficial outcomes in clinical scenarios that could mirror the ones achieved in our training setting.

In clinical settings, both stenting techniques (IVaS and DC) have potential risks associated with the insertion and removal of the stent, particularly the risk of causing vessel damage during these steps. They would be best suited for more challenging cases involving very small or delicate vessels, in the lower range of supermicrosurgery (under 0.5 mm), where traditional techniques may not provide sufficient visibility or protection against wall damage, making stenting techniques necessary. These stenting techniques can significantly enhance outcomes in supermicrosurgical procedures such as lymphedema treatment, perforator flap reconstructions, digital replantations, or revascularizations. Their ability to prevent collapse and maintain vessel integrity during anastomosis makes them ideal for these delicate procedures.

Since the advantage of non-stenting techniques over stenting methods lies in the avoidance of vascular damage during stent manipulation, including sub-visual vessel injuries that can only be detected through electron microscopy, we consider CC a superior approach as it ensures technical feasibility while minimizing the risk of additional vascular damage. The presence of contrast represents the key element in improving the results in supermicrosurgical procedures; therefore, DC is our next technical choice. The use of DC is preferable in vessels with smaller diameters, paying attention to stent manipulation. We believe that the next preferable techniques are IVaS and then finally the classic method.

Our study has some obvious limitations that should be taken into consideration when designing future studies. Firstly, the suture choice (10/0 polypropylene) is not traditionally the most optimal in supermicrosurgery. However, since our study was performed on vessels with a mean diameter of 0.652 mm, which is in the higher range of supermicrovascular structures (under 0.8 mm), we found that the 10/0 suture was more appropriate to handle for a surgeon in training and was necessary in order to assure the replicability and unity of this study, given the availability in our experimental laboratory. Despite not having observed any intimal damage on microscopic visual inspection, our study lacks any quantitative assessment of vessel wall damage, either due to stent manipulation or improper wall passage with the 10/0 suture needle, changes that might occur at a microscopic level at the level of the endothelium. As another limitation, vascular wall damage was decided in our non-living study to be non-applicable, and no pathological microscopic examinations of the vessels were performed. Additionally, even though a biological model is still superior to non-biological models, the non-living chicken wing is inferior to living subjects (either animal or human) due to the lack of biological responses from the vessels, such as spasming, kinking, and the inability to perform in vivo blood flow evaluations or subsequent anastomotic clotting. These factors influence the late evaluation of the patency, which represents the ultimate outcome in a clinical scenario. Another limitation of this study is not including very small vessels with diameters under 0.5 mm, which may change the choice of the stent’s diameter as we aimed to test a uniform stenting method.

Further studies should take into consideration all the aforementioned limitations, as well as include a higher number of anastomoses to improve the statistical significance of the results. Thus, future research could investigate the superiority of CC regarding anastomotic patency and time for completion in the setting of even smaller vessels (under 0.5 mm) and smaller, more appropriate suture sizes (11/0 or 12/0), where stenting techniques are frequently preferred, especially if being performed by more experienced microsurgeons. The marginally improved results we obtained for DC in comparison to IVaS may become statistically significant in such scenarios and could render these future studies more accurate, potentially leading to wider use of the DC method in supermicrosurgery. Furthermore, future studies should take into consideration transitions to living models, such as murine or porcine models, and should incorporate quantitative methods of assessing vessel wall damage in the context of a living model, including histological examination, and the subsequent biological responses such as kinking or spasming.

**Figure 5:**
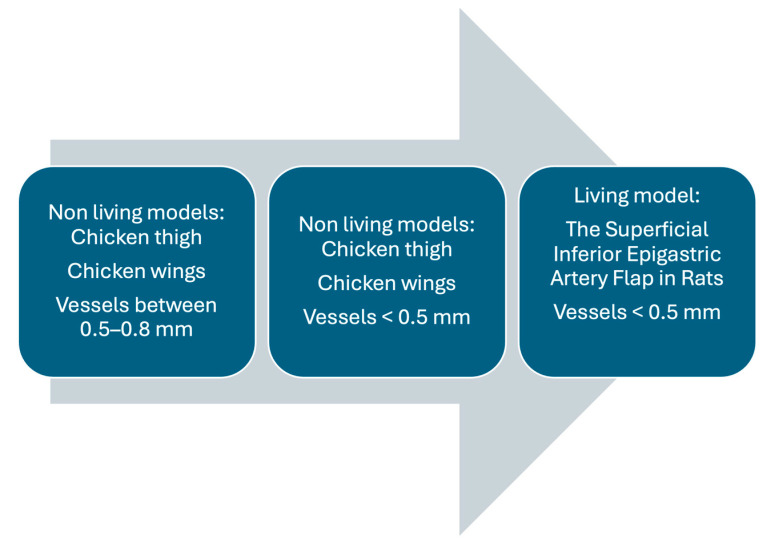
Proposed phased approach for study design and future research directions [26,43].

To address these limitations, we propose a potential design for future research that could build upon the findings presented here. One approach could involve utilizing a non-living model similar to that used by Chen et al. [26] or the one employed in our study, including smaller, more distal vessels. Although this would allow for further refinement and validation of the techniques, it would remain within the constraints of a non-living model. It is crucial to note that such a model inherently limits the ability to assess certain physiological responses. To overcome this, a subsequent phase could involve transitioning to living models, such as rat models [43]. These living models would enable a more comprehensive analysis, including the evaluation of additional factors that influence outcomes and the potential for histopathological examination. By validating the surgical techniques in non-living models first, the transition to living models would be both justified and methodologically robust, facilitating a deeper understanding of the processes under study. This phased approach (Figure 5) would not only address current limitations but also pave the way for a more complete understanding of the research subject in future studies.

## 5. Conclusions

This study explored the technical challenges associated with supermicrosurgery, specifically focusing on microvascular anastomoses and the risk of damaging the vessel wall. This research compares four low-cost and reproducible training models that do not require living subjects, one of them being a new surgical method referred to as the DC technique, which combines intravascular stenting with vascular edge marking. The commonly used CC technique was superior in almost every aspect, including average time for anastomosis completion, patency, and back wall catching. Despite being unable to significantly improve overall anastomotic patency rates in vessels ranging from 0.5 to 0.8 mm, the DC technique provided a reduction in back wall engagement. We anticipate that the DC technique may be favored by trainees in supermicrosurgery on vessels smaller than 0.5 mm, as it enhances surgical precision and helps reduce the learning curve for novice surgeons. This improvement is likely to lead to better outcomes in supermicrosurgical procedures, which demand exceptional skill and accuracy.

## Figures and Tables

**Table 1:** Patency rates and back wall biting for each technique.

Technique	Classic	IVaS	CC	DC	Overall
Number of anastomoses (*n*)	30	30	30	30	120
Patency (*n* (%))	24 (80%)	24 (80%)	30 (100%)	26 (87%)	104 (87%)
Non-patency (*n* (%))	6 (20%)	6 (20%)	0 (0%)	4 (13%)	16 (13%)
No back wall biting (*n* (%))	26 (86.66%)	30 (100%)	30 (100%)	30 (100%)	116 (96.66%)
Back wall biting present (*n* (%))	4 (13.33%)	0 (0%)	0 (0%)	0 (0%)	4 (13.33%)

**Table 2:** The statistical significance when comparing different techniques regarding the patency of anastomosis.

*p*-Values for Patency		Classic	IVaS	CC	DC	Associated Techniques			
						Stenting Techniques (IVaS and DC)	Non-Stenting Techniques (Classic and CC)	Staining Techniques (CC and DC)	Non-Staining (Classic and IVaS)
Classic		-	*p* > 0.05	***p* = 0.009**	*p* > 0.05	*p* > 0.05	-	*p* > 0.05	-
IVaS		*p* > 0.05	-	***p* = 0.009**	*p* > 0.05	-	*p* > 0.05	*p* > 0.05	-
CC		***p* = 0.009**	***p* = 0.009**	-	***p* = 0.03**	***p* = 0.01**	-	-	***p* = 0.008**
DC		*p* > 0.05	*p* > 0.05	***p* = 0.03**	-	-	*p* > 0.05	-	*p* > 0.05
Associated techniques	Stenting techniques (IVaS and DC)	*p* > 0.05	-	***p* = 0.01**	-	-	*p* > 0.05	-	-
	Non-stenting techniques (Classic and CC)	-	*p* > 0.05	-	*p* > 0.05	*p* > 0.05	-	-	-
	Staining techniques (CC and DC)	*p* > 0.05	*p* > 0.05	-	-	-	-	-	***p* = 0.03**
	Non-staining (Classic and IVaS)	-	-	***p* = 0.008**	*p* > 0.05	-	-	***p* = 0.03**	-

**Table 3:** Time required for anastomosis for each technique. Min.—time for the fastest anastomosis; Max.—time for the slowest anastomosis; SD—standard deviation.

Technique	Classic (mm:ss)	IVaS (mm:ss)	CC (mm:ss)	DC (mm:ss)	Overall (mm:ss)
Min.	10:39	13:47	12:45	14:15	10:39
Max.	32:02	28:37	20:35	31:30	32:02
Mean	20:42	21:15	15:40	20:55	19:38
SD	05:14	04:31	01:48	04:22	04:44
Median	20:32	21:07	15:24	20:45	19:01

**Table 4:** Different adaptations of the IVaS technique throughout the literature. N/A—not available; PIST—Preparatory Intravascular Stenting Technique; ICG—indocyanine green; TASS—Temporary Assisting Suspension Suture Technique; DC—Double-Contrast technique.

Author	Year	Technique	Study Type	Subjects Total Number	Vessel Size (mm)	Patency Test	Patency Rate
Chang et al. [31]	2023	Bungee-IVaS	N/A	N/A	N/A	N/A	N/A
Nuri et al. [32]	2023	PIST	Clinical	20	0.36–0.53	ICG lymphangiography	92.8% (For 10 cases with PIST)
Tashiro et al. [33]	2019	Hemi-IVaS	Clinical	34	0.3–0.7	Refill test	100%
Tsumura et al. [34]	2023	Double flipping	Murine models	35	0.35–0.45	N/A	Immediate patency: 90% Late patency: 81%
Wei et al. [29]	1982	TASS	Murine models	24	1.0–1.3	Immediate patency: Refill test Late patency: Dissection	Immediate patency: 100% Late patency: 100%
Our study results	2024	Classic, IVaS, CC, DC	Experimental non-living model (chicken wings)	120	0.5–0.8	“Blue blood” flow	87% (For 30 cases using the DC technique)

## Data Availability

Data are available on request due to restrictions (e.g., privacy, legal, or ethical reasons).
